# Systemic Steroid Application Caused Sudden Death of a Patient with Sudden Deafness

**DOI:** 10.1155/2013/734131

**Published:** 2013-02-11

**Authors:** Eriko Ogino-Nishimura, Takayuki Nakagawa, Ichiro Tateya, Harukazu Hiraumi, Juichi Ito

**Affiliations:** Department of Otolaryngology, Head and Neck Surgery, Graduate School of Medicine, Kyoto University, Kawaharacho 54, Shogoin, Sakyoku, Kyoto 606-8507, Japan

## Abstract

A 63-year-old man, who was diagnosed with sudden sensorineural hearing loss (SSHL), showed severe hypertension 10 hours after prednisolone administration. Subsequently, the patient suddenly died due to pulmonary edema. The autopsy indicated a pheochromocytoma in the right adrenal gland, and the cause of death was determined to be a pheochromocytoma crisis induced by systemic administration of prednisolone. Pheochromocytoma crisis is a life-threatening condition and can result from the use of corticosteroids. Physicians should consider the risk of a pheochromocytoma crisis due to systemic corticosteroids in the treatment of patients with sudden sensorineural hearing loss.

## 1. Introduction

Sudden sensorineural hearing loss (SSHL) is a common condition in which patients lose hearing in 1 ear over the course of 3 days [1]. Systemic corticosteroid administration has been used as a standard therapy for SSHL [1]. On the other hand, the risk of serious complications due to systemic corticoids has been reported [2]. Pheochromocytoma crisis is a serious endocrine emergency that is potentially caused by the systemic administration of corticosteroids to a patient harboring an adrenal incidentaloma [3–6]. The clinical picture of pheochromocytoma crisis is extremely variable, ranging from severe hypertension to circulatory failure and shock, and the condition is often fatal. Herein we report a case of pheochromocytoma crisis that occurred during the treatment of a patient with SSHL. 

## 2. Case Presentation 

**Figure 1:**
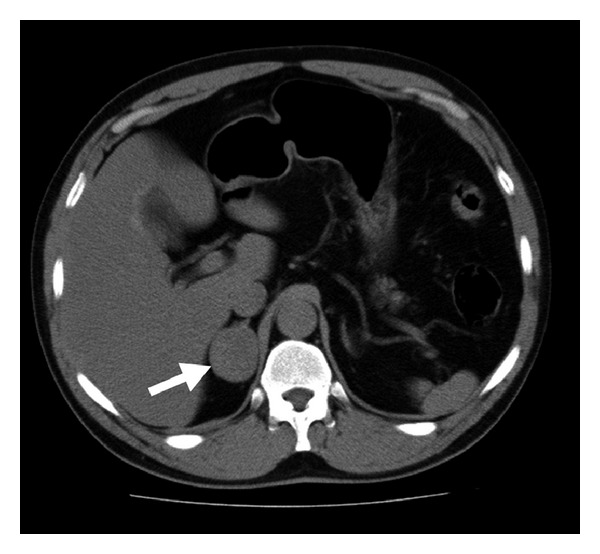
Computed tomography showing a mass in the right adrenal gland (arrow).

In August 2011, a 63-year-old man who initially presented to an ear, nose, and throat clinic with sudden hearing loss of the left ear was diagnosed with sudden sensorineural hearing loss. He was referred to our department because of the presence of comorbidities including diabetes and hypertension. Before the initial administration of medication, we consulted his family doctor regarding the appropriateness of systemic corticosteroids. We were given permission to administer them on the condition that the patient's blood sugar levels were controlled. A recent electrocardiogram of the patient showed no signs of acute myocardial infarction or arrhythmia. His blood pressure on admission was 116/70 mmHg and had been well controlled by a calcium blocker and angiotensin-converting-enzyme inhibitor. General examinations at admission were unremarkable. The patient then received an intravenous injection of 100 mg prednisolone. He complained of dizziness, nausea, and general fatigue 10 hours after prednisolone administration, but did not complain of chest pain, headache, or dyspnea. Hyperglycemia (454 mg/dL), high blood pressure (218/132 mmHg), and moderate tachycardia (119/min) were identified. The hyperglycemia was controlled by an infusion of physiological saline, and his blood pressure decreased to 128/108 mmHg after nausea was controlled with metoclopramide. However, sudden onset of severe dyspnea occurred and the patient experienced cardiopulmonary arrest 1 h later. In spite of resuscitation, the patient died as a result of pulmonary edema. The autopsy indicated a hemorrhagic pheochromocytoma in the right adrenal gland. There were no signs of myocardial infarction or cerebral hemorrhage. A few days later, we learned that an incidental adrenal mass had previously been found in this patient by computed tomography (Figure 1). Consequently, the cause of death was determined as pheochromocytoma crisis induced by prednisolone.

## 3. Discussion

Pheochromocytomas are rare chromaffin cell tumors arising in the adrenal glands that usually produce excess catecholamines leading to paroxysms of hypertension and adrenergic symptoms. A diagnosis of pheochromocytoma is confirmed by the demonstration of elevated 24-hours urinary or plasma catecholamines and their metabolites [3, 4]. However, the most sensitive screening test remains a matter of debate. Pheochromocytoma crisis is a rare, life-threatening condition characterized by deterioration of hemodynamics due to excessive secretion of catecholamines. The crisis can present spontaneously or as a result of unmasking by several factors such as surgery, anesthesia, and drugs, including corticosteroids [4]. The clinical picture of pheochromocytoma crisis is extremely variable, ranging from severe hypertension to circulatory failure and shock, acute myocardial infarction, pulmonary edema, encephalopathy, and multiorgan failure. The primary choice for the treatment of pheochromocytoma crisis is the intravenous application of a nonselective *α*-blocker [3]. The additional use of a *β*-blocker may be needed to prevent tachycardia after controlling blood pressure with an *α*-blocker [3]. 

Including the present case, 15 cases of corticosteroid-induced pheochromocytoma crisis have been reported [4]. The most common presenting symptoms were chest pain, vomiting, and hypertension. In 10 of the 15 patients, serious complications such as congestive heart failure, cardiac arrest, and respiratory failure developed, and in 3 of these patients, the consequences were lethal. The route of corticosteroid administration does not appear to be associated with clinical severity. Pheochromocytoma crisis has been shown to occur even after oral administration of 2 mg dexamethasone [4]. In addition, intervals ranging from 1 to 72 h were observed between corticosteroid administration and crisis onset. 

In this case, systemic prednisolone application for the treatment of SSHL induced the pheochromocytoma crisis. Although systemic corticosteroids are the standard therapy for this condition, the evidence to support their use is weak [1]. Recently, topical corticosteroid treatment has gained considerable attention as an alternative for systemic corticosteroids to avoid serious complications [2, 7]. The effect of topical corticosteroid administration is not inferior to systemic corticosteroid administration [7]. Hence, systemic corticosteroids should be replaced with topical treatment when treating SSHL in patients with relative contraindications to corticosteroids [1, 2, 7]. Incidental adrenal masses must be included among the contraindications for corticosteroid administration.

 In conclusions, we report a case of pheochromocytoma crisis that occurred in a patient during treatment for SSHL. Although pheochromocytoma crisis is rare, it is sometimes lethal. Physicians should be aware of risk of the pheochromocytoma crisis when systemic glucocorticoids are selected for the treatment of SSHL.
